# Regulation of Small GTPase Rab20 by Ikaros in B-Cell Acute Lymphoblastic Leukemia

**DOI:** 10.3390/ijms21051718

**Published:** 2020-03-03

**Authors:** Jonathon L Payne, Chunhua Song, Yali Ding, Pavan Kumar Dhanyamraju, Yevgeniya Bamme, Joseph W Schramm, Dhimant Desai, Arati Sharma, Chandrika Gowda, Sinisa Dovat

**Affiliations:** 1 School of Medicine, Loma Linda University, Loma Linda, CA 92350, USA; jlpayne@llu.edu; 2 College of Medicine, The Pennsylvania State University, Hershey, PA 17033, USA; yding@pennstatehealth.psu.edu (Y.D.); pdhanyamraju@pennstatehealth.psu.edu (P.K.D.); ybamme@pennstatehealth.psu.edu (Y.B.); jschramm@pennstatehealth.psu.edu (J.W.S.); ddesai@pennstatehealth.psu.edu (D.D.); asharma@pennstatehealth.psu.edu (A.S.); cgowda2@pennstatehealth.psu.edu (C.G.); 3 Wexner Medical Center, The Ohio State University, Columbus, OH 43210, USA; chunhua.song@osumc.edu

**Keywords:** Ikaros, CK2, PP1, RAB20, B-ALL

## Abstract

Ikaros is a DNA-binding protein that regulates gene expression and functions as a tumor suppressor in B-cell acute lymphoblastic leukemia (B-ALL). The full cohort of Ikaros target genes have yet to be identified. Here, we demonstrate that Ikaros directly regulates expression of the small GTPase, Rab20. Using ChIP-seq and qChIP we assessed Ikaros binding and the epigenetic signature at the RAB20 promoter. Expression of Ikaros, CK2, and *RAB20* was determined by qRT-PCR. Overexpression of Ikaros was achieved by retroviral transduction, whereas shRNA was used to knockdown Ikaros and CK2. Regulation of transcription from the *RAB20* promoter was analyzed by luciferase reporter assay. The results showed that Ikaros binds the *RAB20* promoter in B-ALL. Gain-of-function and loss-of-function experiments demonstrated that Ikaros represses *RAB20* transcription via chromatin remodeling. Phosphorylation by CK2 kinase reduces Ikaros’ affinity toward the *RAB20* promoter and abolishes its ability to repress *RAB20* transcription. Dephosphorylation by PP1 phosphatase enhances both Ikaros’ DNA-binding affinity toward the *RAB20* promoter and *RAB20* repression. In conclusion, the results demonstrated opposing effects of CK2 and PP1 on expression of Rab20 via control of Ikaros’ activity as a transcriptional regulator. A novel regulatory signaling network in B-cell leukemia that involves CK2, PP1, Ikaros, and Rab20 is identified.

## 1. Introduction

*IKZF1* encodes the 519 amino acid DNA-binding, zinc-finger protein, Ikaros [1,2,3,4]. Ikaros plays a crucial role in regulating normal lymphopoiesis [3,5,6,7] and functions as a tumor suppressor [8]. Germline mutations or deletions that compromise Ikaros activity are associated with the development of B-cell leukemia [9,10,11,12,13,14,15,16], T-cell leukemia, lymphoma [8,17], and primary immunodeficiency [18,19]. Ikaros was shown to regulate myeloid cell proliferation [20,21,22,23,24] and somatic Ikaros alterations are associated with myeloproliferative disorders [25,26,27,28]. Somatic deletion of a single Ikaros allele is associated with pediatric leukemias with resistance to treatment, high relapse rate, and poor prognosis [29,30,31,32,33,34,35,36]. Ikaros mediates its tumor suppressive effects through sequence-specific DNA binding and recruitment of histone remodeling complexes such as NuRD, via direct binding to Mi-2 [37,38,39,40]. Ikaros also directly interacts with and recruits HDAC1 and HDAC2 to the promoters of its target genes [38,41]. These data suggest that Ikaros exerts its tumor suppressive effect through chromatin remodeling at the regulatory elements of its gene targets [42,43,44].

In addition to genetic inactivation, Ikaros activity can be regulated by post-translational phosphorylation and SUMOylation [45,46]. The effect of Ikaros phosphorylation by pro-oncogenic casein kinase II (CK2) has been extensively studied [45]. CK2 is a pleotropic serine/threonine kinase that is overexpressed in multiple cancers including leukemia [47,48]. Studies showed that CK2 directly phosphorylates multiple residues throughout the Ikaros protein [49]. Functional experiments using phosphomimetic and phosphoresistant Ikaros mutants showed that phosphorylation at CK2 phosphosites severely reduces Ikaros’ ability to bind DNA and, thus, in functional inactivation of the Ikaros protein [49]. Pharmacological inhibition of CK2 with small molecule CK2 inhibitors restores Ikaros’ DNA-binding ability along with its tumor suppressor activity and causes leukemia cell cytotoxicity in high-risk patient-derived xenograft (PDX) models of acute lymphoblastic leukemia (ALL) [50]. This discovery highlighted CK2 inhibitors as potential therapeutic agents for high-risk pediatric leukemia [51,52,53].

Ikaros directly interacts with protein phosphatase 1 (PP1) via a PP1-consensus binding site at Ikaros’ C-terminal end [54]. PP1 is a serine/threonine phosphatase that regulates cell division and cell metabolism [55,56,57,58,59,60,61,62]. Ikaros is dephosphorylated by PP1, which reverses the effect of CK2-mediated phosphorylation [54,63]. Mutations at Ikaros’ PP1-interaction site, as well as pharmacological inhibition of PP1, result in Ikaros hyperphosphorylation, severely reduced Ikaros DNA-binding ability, and the loss of Ikaros’ pericentromeric localization. Ikaros’ inability to interact with PP1 also results in increased degradation of Ikaros via the ubiquitin pathway [54]. These data suggest that the balance between CK2 and PP1 plays a crucial role in regulating Ikaros activity and that a perturbation of this balance results in impaired Ikaros function [64,65].

Identification of the genes whose transcription and overall expression are directly regulated by Ikaros provided insights into Ikaros’ function as a regulator of hematopoiesis and a tumor suppressor [66,67,68,69,70,71,72,73]. These studies uncovered an Ikaros regulatory network that controls normal hematopoiesis and malignant transformation [74,75,76,77,78]. Here, we report that Ikaros regulates the expression of the small GTPase Rab20 in leukemia, and that CK2 and PP1 regulate Ikaros’ ability to repress *RAB20* transcription. The results suggested that Ikaros exerts its tumor suppressor activity by regulating *RAB20* expression and demonstrate the role of CK2 and PP1 in regulating the Rab20 signaling pathway.

## 2. Results

### 2.1. Ikaros Binds to the RAB20 Promoter in B-ALL Cells

**Figure 1:**
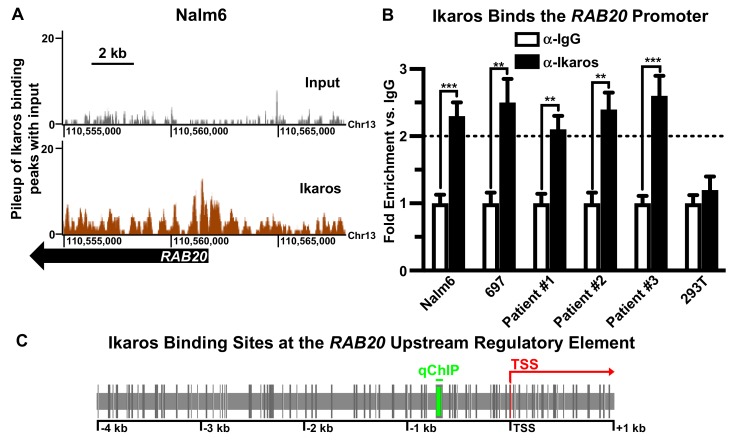
Ikaros binding in the *RAB20* URE. (**A**) ChIP-seq signal map for Ikaros binding to the *RAB20* promoter in the Nalm6 B-ALL cell line. (**B**) qChIP analysis of Ikaros occupancy at the *RAB20* promoter in human B-ALL cell lines, primary human B-ALL cells, and Ikaros-null HEK293T cells. The graphed data is the mean ± SD (error bars) from three independent experiments. (**C**) Schematic diagram of Ikaros consensus binding motifs (–GGGA– and –GGAA–) in the human *RAB20* upstream regulatory element (grey vertical lines). The green line marks the site that was analyzed by qChIP and the TSS is marked by the red arrow.

Determination of global, genome-wide Ikaros DNA occupancy was performed using chromatin immunoprecipitation coupled with next generation sequencing (ChIP-seq). This was performed in the human B-cell acute lymphoblastic leukemia (B-ALL) cell line Nalm6. Analysis of ChIP-seq data identified 5788 prospective target genes regulated by Ikaros in Nalm6 cells [50], including a strong Ikaros binding peak at the promoter sequence of the *RAB20* gene (Figure 1A). *RAB20* was selected for further analysis for several reasons: (1) Analysis of Ikaros occupancy at the *RAB20* promoter by ChIP-seq revealed a strong peak, with the center of the peak located in very close proximity (−104 bp) to the transcriptional start site (TSS) of the *RAB20* gene. This suggests that Ikaros binding likely regulates transcription of *RAB20*; (2) Rab20 most likely has oncogenic activity in colorectal cancer [79,80,81], and myelodysplastic syndrome (MDS) [82,83]. Ikaros functions as a tumor suppressor in these malignancies [20,21,22,23,24,25,26,27,28,81,84]. (3) Rab20 function is associated with increased immune response [85,86,87] and in immune activation of macrophages [88,89]. Ikaros functions as a suppressor of TCR signaling and in macrophages [90,91,92,93]. (4) The potential role of Ikaros in regulating GTPase signaling is unknown and, in general, the mechanisms that regulate transcription of Rab GTPase genes are not well studied; and (5) although Rab20 was suggested to act as an oncogene in various malignancies [79,80,82,94,95,96], its role in B-ALL is unknown. Ikaros binding to the *RAB20* promoter suggests a possible role of Rab20 in B-ALL. Demonstrating the involvement of the CK2–Ikaros axis in regulating *RAB20* expression would represent a new class of genes, GTPases, regulated by Ikaros and provide insight into the cross-talk of signaling pathways in leukemia. Ikaros binding to the promoter of *RAB20* was further confirmed by quantitative chromatin immunoprecipitation (qChIP) in human B-ALL cell lines and primary B-ALL cells (Figure 1B, panels 1–5). Ikaros binding to the *RAB20* promoter was not detected in 293T cells, which do not express Ikaros (Figure 1B, panel 6). A metanalysis of previous ChIP-seq data in patient-derived human pre-B ALL xenograft LAX2 cells, identified strong Ikaros binding at the *RAB20* promoter (Figure S1). A metanalysis of a publicly available B-ALL gene expression dataset identified an inverse correlation between *IKZF1* and *RAB20* expression that was statistically significant (Figure S2).

The *RAB20* gene encodes the Rab20 small GTPase, a protein shown to be involved in regulating cellular proliferation, cell metabolism, and inflammation. DNA sequence analysis of the *RAB20* promoter identified a strong Ikaros consensus binding site located in close proximity to the transcriptional start site of *RAB20* and within the Ikaros peak detected by ChIP-seq. The *RAB20* promoter contains a large number of core Ikaros consensus DNA binding sequences (–GGGA– or –GGAA–) in close proximity to each other (Figure 1C). Since Ikaros binds with higher affinity to DNA sequences that contain two core consensus Ikaros binding sites within 40 bp, these data indicated that the *RAB20* promoter has several high-affinity Ikaros binding sites. This suggests that Ikaros occupancy at the *RAB20* promoter has strong functional significance in cellular metabolism.

### 2.2. Ikaros Represses Transcription of the RAB20 Gene

**Figure 2:**
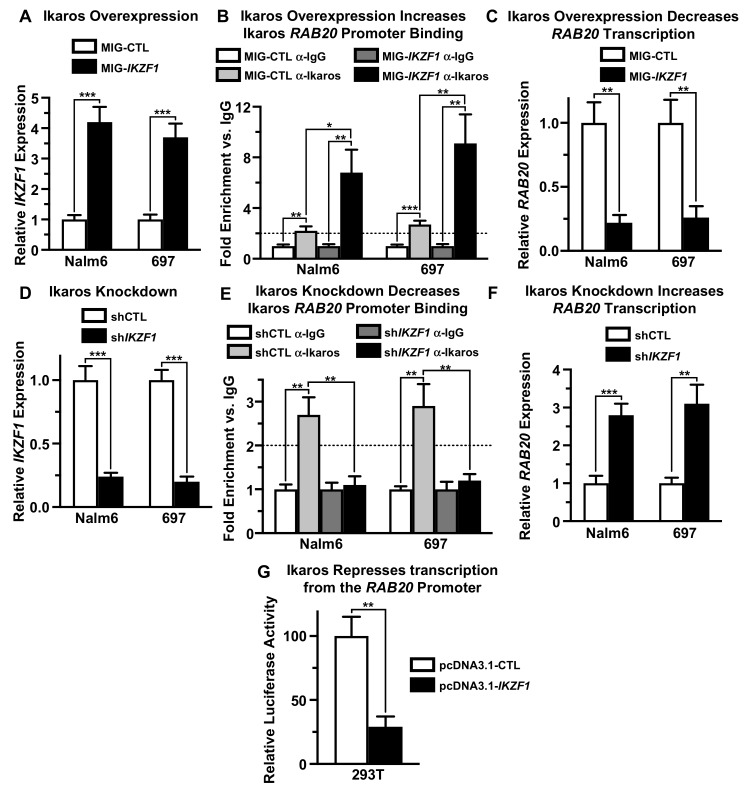
Ikaros represses *RAB20* transcription. (**A**) Human B-ALL cell lines were transduced with empty vector (MIG-CTL) or Ikaros (MIG-*IKZF1*) and relative *IKZF1* expression was determined by qRT-PCR. (**B**) qChIP was used to determine Ikaros occupancy at the *RAB20* promoter after Ikaros overexpression. (**C**) Ikaros overexpression resulted in a decrease in *RAB20* expression as determined by qRT-PCR. (**D**) Human B-ALL cell lines were transduced with scrambled shRNA (shCTL) and shRNA targeting *IKZF1* (sh*IKZF1*) and relative Ikaros expression determined using qRT-PCR. (**E**) qChIP was used to determine Ikaros occupancy at the *RAB20* promoter after Ikaros knockdown. (**F**) Ikaros knockdown resulted in an increase in *RAB20* expression as determined by qRT-PCR. (**G**) The effect of Ikaros on transcription from the URE of human *RAB20* using a luciferase reporter assay following transfection with control vector (pcDNA3.1-CTL) or human Ikaros (pcDNA3.1-*IKZF1*). The graphed data are the mean ± SD (error bars) from three independent experiments.

To establish the functional significance of Ikaros occupancy at the *RAB20* promoter, we evaluated the effect of Ikaros overexpression and knockdown on *RAB20* expression in the human B-ALL cell lines Nalm6 and 697. Overexpression of Ikaros in Nalm6 and 697 cells was achieved by retroviral transduction, using retrovirus that co-expresses Ikaros and green fluorescent protein (GFP) as a marker. Transduction with a retrovirus that does not contain the *Ikaros* gene was used as a negative control. Quantitative PCR analysis (qPCR) showed that retroviral Ikaros transduction results in increased Ikaros expression compared to the negative control in both Nalm6 and 697 cells (Figure 2A). Increased Ikaros expression was associated with increased Ikaros binding to the promoter of the *RAB20* gene by qChIP compared with the negative control (Figure 2B). Increased Ikaros binding to the *RAB20* gene was associated with strong transcriptional repression of *RAB20* per qPCR compared with the negative control (Figure 2C). These data suggest that Ikaros transcriptionally represses the *RAB20* gene.

Ikaros loss-of-function was achieved using Ikaros shRNA. Ikaros knock-down with shRNA resulted in reduced Ikaros expression in Nalm6 and 697 cells compared with negative controls (Figure 2D). Reduced Ikaros expression was associated with diminished Ikaros occupancy at the *RAB20* promoter, as evidenced by qChIP (Figure 2E). Loss of Ikaros binding to the *RAB20* promoter was associated with increased expression of *RAB20* in both Nalm6 and 697 B-ALL cells (Figure 2F). Together with data from experiments described in Figure 2A–C, these results demonstrated that Ikaros negatively regulates expression of *RAB20* in B-ALL cells by acting as a transcriptional repressor at the *RAB20* promoter.

We tested whether Ikaros can directly repress transcription of the *RAB20* gene using the luciferase reporter assay. 293T cells were co-transfected with plasmid expressing the luciferase gene under control of the *RAB20* promoter along with the Ikaros-expressing plasmid or the empty vector (negative control). Luciferase expression was determined using the luciferase reporter assay. The results showed that co-transfection with Ikaros significantly reduces transcription from the *RAB20* promoter compared with the negative control (Figure 2G). These results demonstrated that Ikaros can repress transcription by directly binding to the promoter of the *RAB20* gene.

### 2.3. Ikaros Represses RAB20 Transcription via Chromatin Remodeling

Next, we sought to determine the mechanism through which Ikaros represses transcription of *RAB20* in B-ALL. One of the main mechanisms through which Ikaros represses its target genes involves chromatin remodeling and the formation of facultative heterochromatin [97]. We determined how Ikaros gain-of-function and loss-of-function affect the chromatin signature at the *RAB20* promoter in Nalm6 and 697 cells.

**Figure 3:**
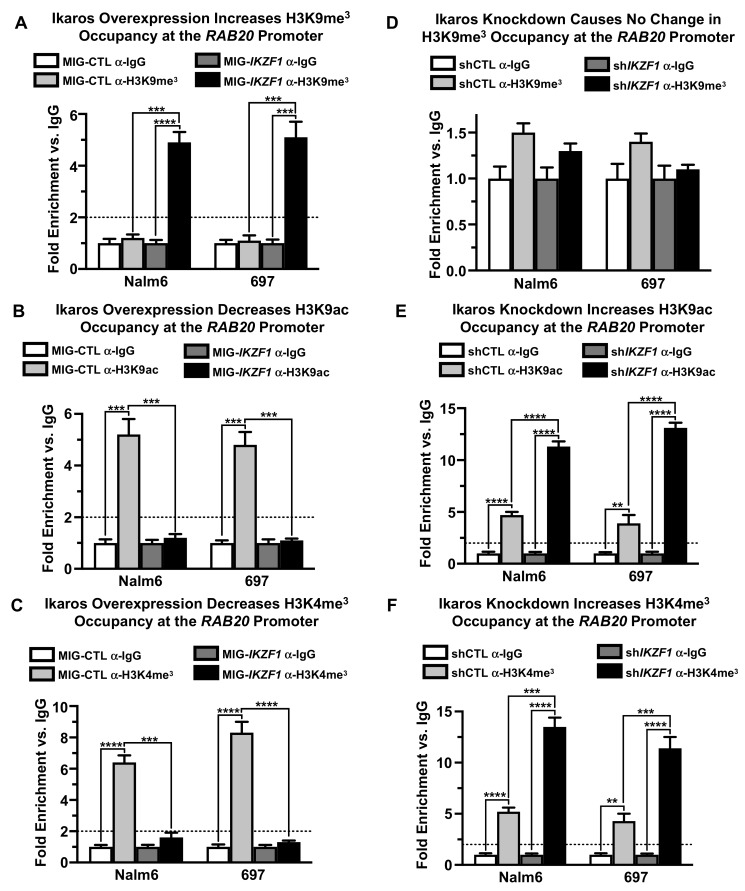
Ikaros increases the occupancy of closed chromatin markers at the *RAB20* promoter. **(A–C**) qChIP was used to determine relative H3K9me^3^, H3K9ac, and H3K4me^3^ occupancy at the *RAB20* promoter after Ikaros overexpression (MIG-*IKZF1*) compared with the control (MIG-CTL). (**D–F**) qChIP was used to determine relative H3K9me^3^, H3K9ac, and H3K4me^3^ occupancy at the *RAB20* promoter after Ikaros knockdown (sh*IKZF1*) compared with a scrambled control (shCTL). The graphed data are the mean ± SD (error bars) from three independent experiments.

H3K9ac and H3K4me3 are the epigenetic markers of open and active chromatin, whereas H3K9me3 is a marker of closed and repressive chromatin. We found that overexpression of Ikaros by retroviral transduction results in enrichment in H3K9me3 histone modification markers at the promoter of the *RAB20* gene in both Nalm6 and 697 cells compared with the negative control (Figure 3A). This was also associated with a loss of H3K9ac and H3K4me3 markers at the *RAB20* promoter (Figure 3B,C). Ikaros knockdown with shRNA resulted in an absence of the H3K9me3 marker (Figure 3D) at the promoter of the *RAB20* gene along with increased H3K9ac and H3K4me3 markers (Figure 3E,F) at the *RAB20* promoter.

Additional analyses showed no enrichment of HDAC1 histone deacetylase at the *RAB20* promoter and that alterations of Ikaros expression do not affect HDAC1 occupancy around the *RAB20* transcriptional start site (data not shown). These results suggested that HDAC1 is not involved in the regulation of *RAB20* transcription.

Overall, these results suggested that Ikaros represses transcription of the *RAB20* gene by inducing the formation of repressive chromatin at the *RAB20* promoter via an HDAC1-independent mechanism.

### 2.4. Casein Kinase II (CK2) Regulates Ikaros’ Ability to Repress RAB20 Transcription

**Figure 4:**
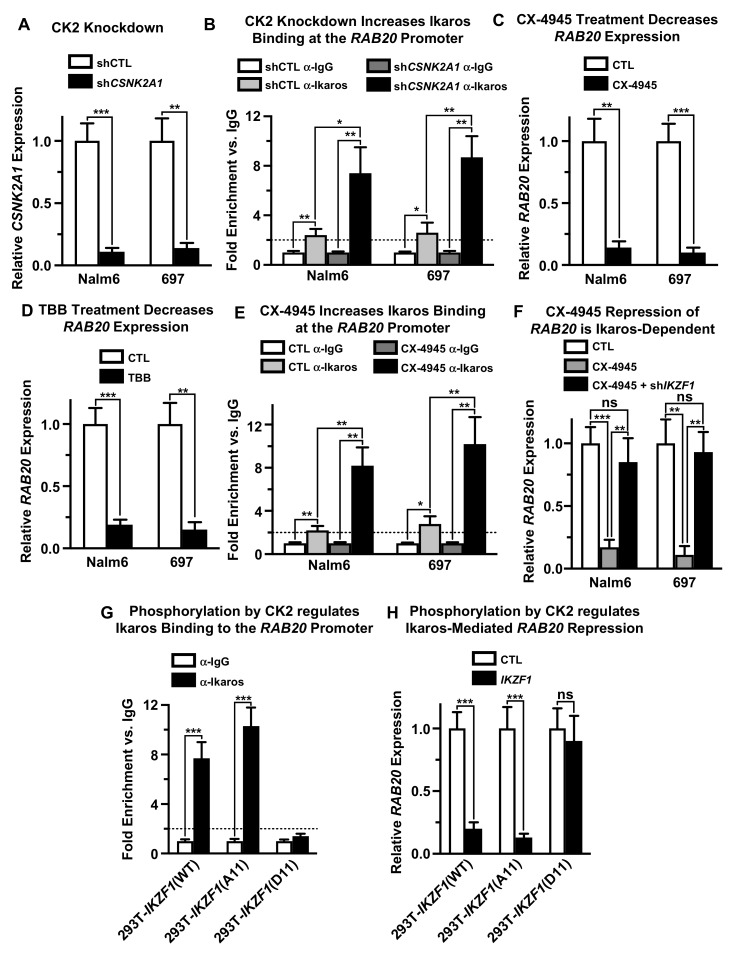
CK2 regulates Ikaros’ ability to repress *RAB20* transcription. (**A**) Human B-ALL cell lines were transduced with scrambled shRNA (shCTL) and shRNA targeting CK2α (sh*CSNK2A1*) and relative *CSNK2A1* expression was determined using qRT-PCR. (**B**) qChIP was used to analyze Ikaros occupancy at the *RAB20* promoter after CK2α knockdown (sh*CSNK2A1*). (**C,D**) Human B-ALL cell lines were treated with pharmacological CK2 inhibitors CX-4945 and TBB, and *RAB20* expression was analyzed using qRT-PCR. (**E**) qChIP was used to analyze Ikaros occupancy at the *RAB20* promoter after treatment with CX-4945. (**F**) Human B-ALL cell lines were treated with CK2 inhibitor CX-4945 and transduced with shRNA targeting Ikaros (sh*IKZF1*) and *RAB20* expression was analyzed by qRT-PCR. (**G**) HEK293T cells were transduced with wild-type Ikaros (*IKZF1*-(WT)), Ikaros with phosphoresistant alanine mutations at CK2 phosphosites (*IKZF1*-(A11)), and Ikaros with phosphomimetic aspartate mutations at CK2 phosphosites (*IKZF1*-(D11)). qChIP was used to determine Ikaros occupancy at the *RAB20* promoter. (**H**) qRT-PCR was used to determine *RAB20* expression in HEK293T cells transduced with wild-type Ikaros (*IKZF1*-(WT)) and Ikaros phosphoresistant *IKZF1*-(A11) and phosphomimetic *IKZF1*-(D11) mutants at CK2 phosphosites. The graphed data are the mean ± SD (error bars) from three independent experiments.

Casein kinase II (CK2) directly phosphorylates Ikaros at multiple evolutionarily-conserved serine and threonine residues and that phosphorylation by CK2 can regulate Ikaros’ DNA-binding affinity and its ability to function as a transcriptional regulator of its target genes [49]. We tested whether CK2 regulates Ikaros’ function as a transcriptional repressor of *RAB20*. Molecular inhibition of CK2 by knock-down with shRNA directed against the CK2 α catalytic subunit (*CSNK2A1*), resulted in reduced expression of CK2 (Figure 4A). This was associated with increased Ikaros binding to the promoter of the *RAB20* gene in both Nalm6 and 697 cells, as evidenced by qChIP (Figure 4B), and with profoundly reduced transcription of *RAB20* in both cell lines (Figure 4C). Pharmacological inhibition of CK2 using its specific inhibitor, CX-4945, as well as with a structurally-different CK2 inhibitor, TBB, also resulted in a strong negative effect on *RAB20* transcription in both B-ALL cell lines (Figure 4D). Treatment with CX-4945 resulted in increased Ikaros binding to the *RAB20* promoter, as evidenced by the qChIP assay (Figure 4E). These data showed that CK2 inhibition negatively regulates *RAB20* transcription and suggested that the mechanism of CK2-mediated repression of *RAB20* involves transcriptional repression by Ikaros, along with the increased DNA binding of Ikaros to the promoter of the *RAB20* gene.

CK2 phosphorylates many proteins. We tested whether Ikaros activity is essential for CK2-mediated repression of *RAB20* in human B-ALL cells. The results showed that knock-down of Ikaros with shRNA, abolishes CK2-induced transcriptional repression of the *RAB20* gene in both Nalm6 and 697 cells (Figure 4F). These results demonstrated that negative regulation of *RAB20* expression by CK2 inhibition occurs via Ikaros transcriptional repression activity and, thus, Ikaros activity is critical for CK2-mediated regulation of *RAB20* transcription.

Next, we tested whether phosphorylation of the known CK2 phosphosites on the Ikaros protein regulates its ability to repress transcription of the *RAB20* gene. For these experiments, we used Ikaros phosphomimetic and phosphoresistant mutants that were previously described [49]. The Ikaros phosphomimetic mutant (*IKZF1*-D11) has 11 serine or threonine residues that are known to be phosphorylated in vivo by CK2, mutated into aspartate. In contrast, the Ikaros phosphoresistant mutant (*IKZF1*-A11) has the same 11 serine of threonine residues mutated into alanine. 293T cells that do not express endogenous Ikaros were transfected with wild-type Ikaros or with Ikaros phosphomimetic or phosphoresistant mutants and the DNA binding of Ikaros proteins to the promoter of the endogenous *RAB20* gene and the effect of each protein on *RAB20* transcription was analyzed by qChIP and qRT-PCR, respectively. The results showed that both wild-type Ikaros (*IKZF1*-WT) and its phosphoresistant mutant (*IKZF1*-A11) bind to the *RAB20* promoter with high affinity in 293T cells, although the DNA-binding affinity of *IKZF1*-A11 appears to be somewhat stronger compared to that of the wild-type Ikaros (Figure 4G). However, the Ikaros phosphomimetic mutant (*IKZF1*-D11) bound poorly to the promoter of the *RAB20* gene in 293T cells as measured by qChIP (Figure 4G). Both wild-type and the phosphoresistant Ikaros mutant (*IKZF1*-A11) resulted in repression of *RAB20* gene transcription in 293T cells, whereas the Ikaros phosphomimetic mutant (*IKZF1*-D11) had no effect on the expression of the *RAB20* genes, as measured by qRT-PCR (Figure 4H). These data demonstrated that CK2 regulates Ikaros’ ability to bind to the promoter of the *RAB20* gene and transcriptionally repress its expression by directly phosphorylating Ikaros proteins on one or several serine/threonine residues. These results thus establish that *RAB20* transcription is regulated by the CK2–Ikaros signaling axis.

### 2.5. Protein Phosphatase 1 (PP1) Regulates Ikaros’ Function as Repressor of RAB20 Transcription

**Figure 5:**
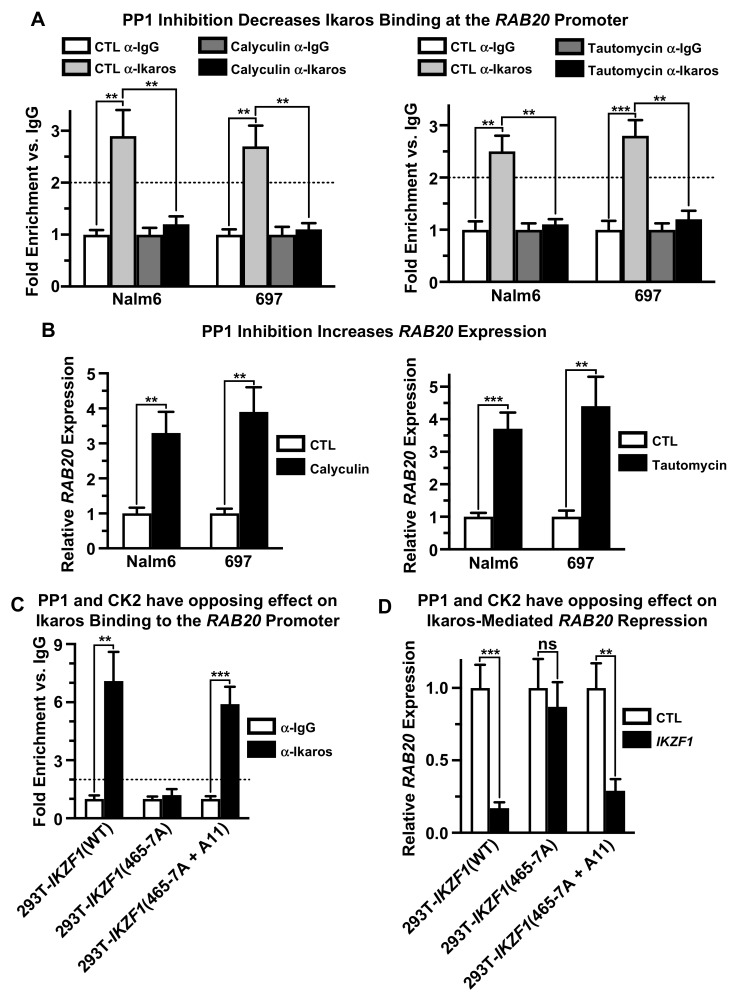
PP1 regulates repression of *RAB20* by Ikaros. (**A**) Human B-ALL cell lines were treated with pharmacological PP1 inhibitors calyculin and tautomycin. qChIP was used to determine Ikaros occupancy at the *RAB20* promoter. (**B**) *RAB20* expression was analyzed using qRT-PCR after PP1 inhibition with calyculin and tautomycin. (**C**) HEK293T cells were transduced with wild-type Ikaros (*IKZF1*(WT)), Ikaros with the mutated PP1 binding-site (*IKZF1*(565-7A)), and Ikaros with the PP1 binding-site mutated and phosphoresistant alanine mutations at CK2 phosphosites (*IKZF1*(465-7A + A11)). qChIP was used to determine Ikaros binding at the *RAB20* promoter. (**D**) qRT-PCR was used to determine *RAB20* expression in HEK293T cells transduced with wild-type Ikaros (*IKZF1*-(WT)) and mutated Ikaros (*IKZF1*-(465-7A) and *IKZF1*-(465-7A + A11)). The graphed data are the mean ± SD (error bars) from three independent experiments.

We tested the effect of phosphatases on Ikaros’ ability to repress transcription of the *RAB20* gene. Treatment of both Nalm6 and 697 cells with a strong inhibitor of protein phosphatase 1 (PP1) and protein phosphatase 2A (PP2A), calyculin, results in the loss of Ikaros binding to the promoter of the *RAB20* gene as measured by qChIP (Figure 5A, left panel). A similar effect was achieved when Nalm6 and 697 cells were treated with tautomycin, which is a highly selective inhibitor of PP1 (Figure 5A, right panel). Treatment with both calyculin and tautomycin resulted in increased transcription of the *RAB20* gene in both Nalm6 and 697 cells, as measured by qRT-PCR (Figure 5B). These data showed that dephosphorylation of Ikaros is essential for its function as a transcriptional repressor of the *RAB20* gene in B-ALL cells.

Ikaros is a substrate for PP1. The Ikaros protein contains a consensus binding site for PP1, which is essential for Ikaros-PP1 interaction and for dephosphorylation of Ikaros by PP1 [54]. Since the treatment of B-ALL cells with a specific PP1 inhibitor, tautomycin, strongly affects Ikaros binding to the *RAB20* promoter and repression of *RAB20* transcription, we tested whether the interaction between Ikaros and PP1 affects the ability of Ikaros to repress *RAB20*. The wild-type Ikaros or the Ikaros mutant that contains alanine point mutations at amino acids 465 and 467 that disrupt Ikaros–PP1 interactions (*IKZF1*-465-7A) was transfected into 293T cells, and the effect of the Ikaros–PP1 interaction on Ikaros’ function in regulating *RAB20* expression was analyzed. The results showed that inhibition of PP1 interaction with Ikaros (*IKZF1*-465-7A) results in the loss of Ikaros binding to the promoter of the *RAB20* gene compared to the wild type Ikaros, as measured by qChIP (Figure 5C panels 1 and 2). Unlike wild-type Ikaros, the *IKZF1*-465-7A mutant had no effect on *RAB20* transcription (Figure 5D, panels 1 and 2). These data showed that PP1 regulates *RAB20* expression, and that the direct interaction of Ikaros with PP1 is essential for Ikaros binding to the promoter of the *RAB20* gene and for repression of *RAB20*.

We tested whether dephosphorylation of the CK2 phosphosites on the Ikaros protein is critical for the effect of PP1 on *RAB20* expression. The effect of an Ikaros mutant that is unable to interact with PP1 but which also has phosphoresistant mutations at CK2 phosphosites (*IKZF1*-465-7A+A11) was evaluated for its effect on *RAB20* transcription. The results showed that phosphoresistant mutations at CK2 phosphosites restored the DNA-binding ability of the Ikaros mutant that did not interact with PP1 (Figure 5C, panel 3) as well as Ikaros’ function as a transcriptional repressor of the *RAB20* gene (Figure 5D, panel 3). Together, these results demonstrated that direct dephosphorylation of CK2 phosphosites on Ikaros by PP1 is essential for Ikaros binding to the promoter of the *RAB20* gene and its transcriptional repression. These data demonstrated that regulation of *RAB20* transcription by Ikaros is regulated in opposing ways by CK2 and PP1.

## 3. Discussion

Ikaros’ role in hematopoiesis, immune response, and tumor suppression depends on its ability to regulate expression of its target genes. The gain-of-function and loss-of-function experiments presented above demonstrated that Ikaros regulates expression of the *RAB20* gene in B-cell acute lymphoblastic leukemia (B-ALL). Ikaros regulates *RAB20* transcription both directly, as evidenced by the luciferase reporter assay, and via chromatin remodeling by inducing the formation of heterochromatin at the *RAB20* promoter, characterized by the H3K9me3 repressive marker.

Rab20 belongs to the Rab family of over 60 small GTPase genes [98]. Proteins encoded by these genes are critical for regulating membrane trafficking [99] and vesicular transport in all cells in eukaryotes [100,101]. Numerous reports demonstrated the important role of the Rab20 protein in immune response [85,86,87]. Rab20 is highly expressed in macrophages, where it plays a role in phagosome biology [102,103] and regulation of endosomal morphology [88]. Expression of Rab20 in macrophages is positively regulated by the NF-κB pathway following mycoplasma infection [89], as well as by interferon-γ (IFN-γ) treatment [88], suggesting the role of Rab20 in immune activation of macrophages. Deregulation of *RAB20* expression was associated with various types of human malignancies: *RAB20* overexpression was pronounced in pancreatic cancer cell lines and in primary pancreatic carcinoma [94], in exocrine pancreatic carcinoma, and in preneoplastic pancreatic tissue compared with normal pancreatic cells [94]. RAB20 amplifications were strongly correlated with the presence of high-grade dysplastic colorectal adenomas, and aberrations of Rab20 were associated with high-risk colorectal adenomas [79]. Amplification of Rab20 was strongly correlated with adenoma recurrence and the presence of colorectal carcinomas [79], whereas overexpression of *RAB20* was associated with colorectal carcinoma liver metastases [80]. Deregulation and a potential oncogenic role for Rab20 was reported in triple negative breast cancer [95] and in bladder cancer [96]. The potential role of Rab20 in leukemia is not well studied, although overexpression of *RAB20* was observed in myelodysplastic syndrome [82]. Since Ikaros’ role in macrophage function [104,105,106], colorectal cancer [81], and MDS [83] was described previously, the data presented here suggested that Ikaros activity in these conditions might be mediated via regulation of *RAB20* transcription. Ikaros’ role in regulating GTPases was previously unexplored, but the data presented here demonstrated the involvement of the CK2-Ikaros axis in regulating expression of the Rab20 GTPase in B-ALL. These data provide new insights into the cross-talk of signaling pathways in leukemia.

Ikaros is phosphorylated at multiple amino acids [107]. Phosphorylation was reported to regulate Ikaros’ DNA-binding affinity, which is essential for its function as a transcriptional regulator [107,108]. CK2 phosphorylates Ikaros at evolutionarily-conserved serine/threonine residues, and thus directly regulates Ikaros’ ability to bind promoters of its target genes, to localize to pericentromeric heterochromatin, and to function as a regulator of gene expression [64,66,67,68,69,70,71,72,109,110]. Increased expression of CK2 was associated with various types of leukemia as well as with other human malignancies. Overexpression of CK2 in leukemia results in reduced Ikaros DNA-binding affinity, and its function as a transcriptional regulator and tumor suppressor; thus, loss of Ikaros function due to CK2 overexpression has a leukemogenic effect [65,111]. The data presented in this manuscript demonstrate that CK2 is a critical regulator of Ikaros’ ability to bind the *RAB20* promoter and control *RAB20* transcription. Both molecular and pharmacological inhibition of CK2 as well as phosphoresistant mutations at CK2 phosphosites in the Ikaros protein enhanced Ikaros binding to the *RAB20* promoter and transcriptional repression of Rab20. These results suggested that the CK2 signaling pathway can affect membrane trafficking in leukemia cells through regulation of Rab20 expression via Ikaros.

**Figure 6:**
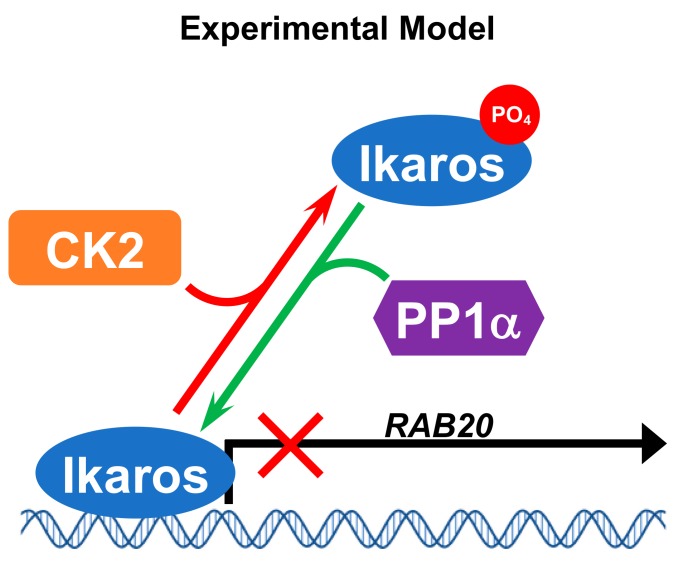
Model of proposed regulation of Rab20 expression by Ikaros, CK2, and PP1α. Ikaros represses *RAB20* transcription. PP1α stimulates Ikaros activity resulting in decreased *RAB20* transcription. CK2 inhibits Ikaros activity resulting in increased Rab20 expression.

Protein phosphatase 1 can function as a tumor suppressor in human malignancies [58,59,60,61]. PP1 was reported to directly bind the PP1 consensus binding site at the C-terminal end of the Ikaros protein. The role of PP1 in the control of Ikaros function in regulating gene expression has not been extensively studied, and the transcription of only one gene, terminal deoxynucleotidetransferase (*TdT*), was reported to be regulated by PP1 via Ikaros dephosphorylation [63]. Dephosphorylation of Ikaros by PP1 increases Ikaros’ DNA-binding affinity, its protein stability, and its ability to function as a transcriptional regulator [54]. The presented data using mutant Ikaros that is unable to interact with PP1 showed that PP1 is a critical regulator of Ikaros’ ability to control *RAB20* transcription and that PP1 directly counteracts CK2 effects on *RAB20* transcription. Thus, the presented data demonstrate the opposing effects of CK2 and PP1 on Rab20 function via control of Ikaros activity as a regulator of Rab20 expression (Figure 6).

In summary, the results presented in this manuscript identify a novel regulatory network in leukemia that involves CK2, PP1, Ikaros and the small GTPase Rab20. The data revealed an intersection of CK2 kinase and PP1 phosphatase signaling via phosphorylation and/or dephosphorylation of the Ikaros tumor suppressor and show that CK2 and PP1 signal transduction pathways have opposing effects on the expression of the Rab20 small GTPase. The results presented here provide novel insights into signaling networks that regulate tumor suppression in B-cell acute lymphoblastic leukemia.

## 4. Materials and Methods

### 4.1. Cells and Cell Culture

Nalm6 and 697 B-cell acute lymphoblastic leukemia (B-ALL) cell lines were obtained from the American Type Culture Collection (ATCC) and the German Collection of Microorganisms and Cell Cultures (DSMZ), respectively. Primary B-ALL leukemia cells were obtained from Loma Linda University (Loma Linda, CA, USA). These samples were taken from patients and de-identified by stripping them of all direct patient identifiers. Their use was approved by the institutional review boards at Loma Linda University and the Penn State College of Medicine. All of the cell lines and patient samples used in this study expressed wild-type *IKZF1*. Nalm6 and 697 cell lines were cultured in RPMI 1640 medium (Corning) supplemented with 0.03% L-glutamine and 10% fetal bovine serum (Hyclone) (RPMI 1640–10). Cells were incubated at 37 °C in a humidified atmosphere of 5% CO_2_ overnight. Primary leukemia cell lines were briefly cultured (1 day) in the same conditions prior to in vitro experimentation.

### 4.2. Metanalyses

Ikaros binding at the *RAB20* promoter in patient-derived human pre-B ALL xenograft cells, LAX2, was determined by analyzing genome-wide Ikaros ChIP-seq data made previously available by H. Schjerven and M. Muschen on the Gene Expression Omnibus (GEO) under access number GSE58825 [112]. The GEO contributing authors noted that LAX2 expresses wild-type, full-length Ikaros. The correlation between *IKZF1* and *RAB20* expression in patients with B-ALL was determined by analyzing gene expression datasets made publicly available by ML den Boer and JM Boer on the Gene Expression Omnibus under access number GSE87070 [113].

### 4.3. Reagents

4,5,6,7-tetrabromobenzotriazole (TBB) (Sigma), CX-4945 (MedChem Express), calyculin A (CalBiochem), and tautomycin (EMD Millipore) were dissolved in dimethylsulfoxide (DMSO) (Sigma) to create stock solutions. Control cell lines were dosed with a volume of DMSO equivalent to the volume received by the cells treated with the highest concentration of drug.

### 4.4. CaPO_4_ Transfection and 293T Gene Expression

Lenti-X 293T cells (Clontech) were grown in DMEM (Corning) supplemented with 4.5% glucose, 0.06% L-glutamine, 0.01% sodium pyruvate, and 10% heat-inactivated fetal bovine serum (Hyclone) (DMEM–10). 293T cells were trypsinized with 1.0 mL of 0.25% trypsin (Corning) at 90% confluency and 2.0 × 10^6^ cells were resuspended in 9.0 mL of DMEM-10, plated in a 10 cm tissue culture dish, and incubated at 37 °C in a humidified atmosphere of 5% CO_2_ overnight. For one 10 cm dish of 293T cells, 20 μg of transfection-grade pcDNA3.1 expression plasmid (prepared using the Macherey–Nagel Nucleobond Xtra Maxi kit) was diluted with 100 μL of freshly-prepared, sterile-filtered 2.0 M CaCl_2_ (Sigma). This mixture was then diluted to 500 μL with sterile, DNase-free water. We added 500 μL of 0.22 μm filtered 2× Hepes-buffered saline, pH 7.05, dropwise to the DNA-CaCl_2_ suspension with constant agitation. This suspension was incubated at room temperature for 10 min before being added dropwise to the plated Lenti-X 293T cells, which were returned to the cell culture incubator. After 24 h, the transfection media was aspirated and the cells were supplied with 10 mL of fresh DMEM-10. 293T cells were then grown for an additional 48 h before harvesting total RNA or formaldehyde crosslinking for chromatin immunoprecipitation.

### 4.5. Expression Plasmids

The construction of the expression plasmids used in this study were described previously: pcDNA3.1-*IKZF1*(WT), pcDNA3.1-*IKZF1*(A11), and pcDNA3.1-*IKZF1*(D11) in [49]; pcDNA3.1-*IKZF1*(465-7A) and pcDNA3.1-*IKZF1*(465-7A + A11) in [54]; and pMIG-CTL & pMIG-*IKZF1*(WT) in [114].

### 4.6. Luciferase Reporter Assay

The luciferase reporter assay experiment was performed as previously described [115]. The pROM-RAB20 vector was purchased from Switchgear Genomics (S719730) and incorporated the *RAB20* promoter from bases –847 to +59 relative to the *RAB20* transcription start site (TSS).

### 4.7. Retroviral Transduction

Retroviruses were produced by transient transfection in amphotropic packaging 293 cell lines as described previously [49] using pMIG-CTL or pMIG-*IKZF1*(WT). Nalm6 and 697 cell lines were plated in 24-well plates at 4 × 10^5^ cells/well and suspended in retroviral supernatants with 10 μg/mL polybrene and centrifuged at 1000× *g* at 37 °C for 2 h. The cells were then suspended in fresh RPMI 1640–10 and cultured in a humidified incubator at 37 °C and 5% CO_2_ for 3 days. The GFP+ cells were then sorted using a FACS Aria SORP (Becton Dickinson) instrument in the Penn State College of Medicine’s Flow Cytometry Core. Sorted cells were further cultured using the above conditions.

### 4.8. Ikaros and CK2 shRNA Knockdown

Ikaros (*IKZF1*) and *CSNK2A1* (CK2α) knockdown in Nalm6 and 697 cells was accomplished using pGP-V-RS shRNA plasmids (Origene) and a neon electroporation system (ThermoFisher Scientific) as described previously [115]. Knockdown of Ikaros and CK2α was confirmed using qRT-PCR.

### 4.9. Quantitative RT-PCR (qRT-PCR)

RNA isolation, cDNA generation, and qPCR conditions were previously described [115]. The following primers were used in this study: 18s RNA: GTAACCCGTTGAACCCCATT (sense) and CCATCCAATCGGTAGTAGCG (antisense); *RAB20*: CGCCAAGACCGGCTACAAT (sense) and GGCACCACCAGGTCAAAGAG (antisense); *CSNK2A1* (CK2α): AGCGATGGGAACGCTTTG (sense) and AAGGCCTCAGGGCTGACAA (antisense); and *IKZF1*: GGCGCGGTGCTCCTCCT (sense) and TCCGACACGCCCTACGACA (antisense).

### 4.10. qChIP

qChIP assays for Ikaros binding in B-ALL cell lines were previously described [116]. qChIP assays for H3K4me3, H3K9me3, and H3K9ac histone markers were performed according to the same protocol as Ikaros qChIP except using 1 × 10^7^ cells and 1–5 μg of antibody for the histone modification markers. The qChIP primers used to link immunoprecipitated DNA to the *RAB20* promoter were: AGCGCCCCCATCTCTAATCT (sense) and AATGAGCGTCTGCGGAACTC (antisense).

### 4.11. ChIP-Seq Experiments

ChIP-seq assays for Ikaros binding in Nalm6 cells were performed as previously described [115]. Alignment of Ikaros binding peaks with the human genome was performed by the Genome Sciences Facility at the Penn State College of Medicine. ChIP-seq data are accessible on Gene Expression Omnibus using access number GSE44218.

### 4.12. Antibodies

The antibodies used to immunoprecipitate and detect the C terminus of Ikaros (Ikaros-CTS) have been described previously [117]. The antibodies used for chromatin immunoprecipitation for epigenetic changes were as follows: anti-rabbit IgG (ab46540, Abcam), anti-H3K4me3 (ab8580, Abcam), anti-H3K9me3 (ab8898, Abcam), and anti-H3K9ac (ab4441, Abcam).

### 4.13. Statistics

P-value summaries were as follows: P > 0.05 (ns); P ≤ 0.05 (*); P < 0.01 (**); P < 0.001 (***); P < 0.0001 (****). Statistics were performed in GraphPad Prism 8. A two-tailed, unpaired *t*-test with Welch’s correction was used for Figure 2G. The statistical analyses on all other panels were determined using multiple two-tailed *t*-tests. Statistical significance was determined using the Holm–Sidak method with α = 0.05. Each row (representing a cell line or primary cell) was analyzed individually, without assuming a consistent SD. The number of *t*-tests per analysis varied based on the number of cell lines or primary cells analyzed per graph. Statistical analysis was not performed on qChIP values where the signal was less than two-fold greater than the background (α-IgG) level as binding below this level was deemed biologically insignificant. This threshold is denoted by a dotted line on all graphs containing qChIP data where permitted by the range of the y axis.

## Supplementary Materials

Supplementary materials can be found at https://www.mdpi.com/1422-0067/21/5/1718/s1.

## Abbreviations

B-ALL	B-Cell Acute Lymphoblastic Leukemia
ChIP-seq	Chromatin Immunoprecipitation and Sequencing
GEO	Gene Expression Omnibus
PDX	Patient-Derived Xenograft
qChIP	Quantitative Chromatin Immunoprecipitation
qRT-PCR	Quantitative Reverse Transcription Polymerase Chain Reaction
TSS	Transcription Start Site
