# Supplementary material for: Regulation of Small GTPase Rab20 by Ikaros in B-Cell Acute Lymphoblastic Leukemia
Source: Int J Mol Sci. 2020 Mar 3;21(5):1718. doi: 10.3390/ijms21051718 (PMC7084408; doi:10.3390/ijms21051718)
Supplement: Supplementary file 1 [file ijms-21-01718-s001.pdf]

Ikaros Binds the *RAB20* Promoter in Primary B-ALL
(Metanalysis of GEO data contributed by H. Schjerven & M. Muschen)

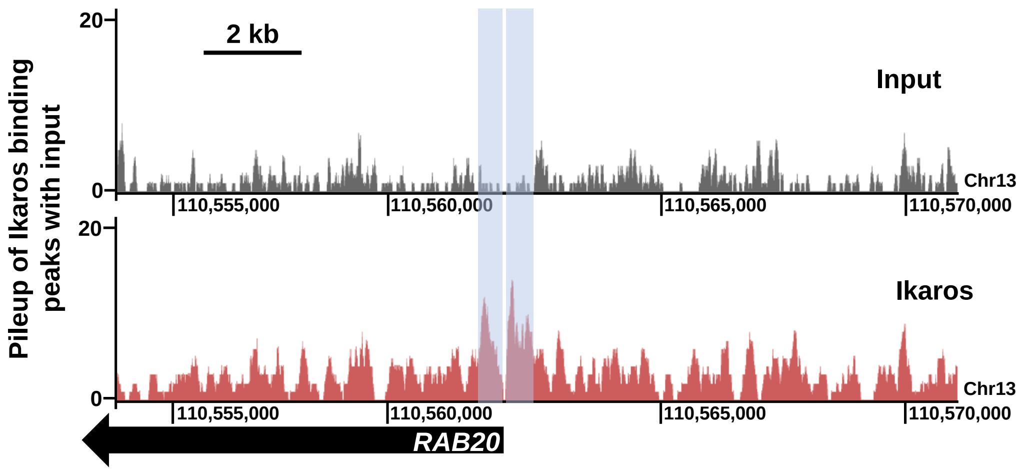

Supplemental Figure 1. Ikaros binds the *RAB20* promoter in Primary B-ALL. Metanalysis of ChIP-seq signal in LAX2 primary Pre-B ALL cells showed Ikaros binding to the *RAB20* promoter. Ikaros binding peaks at promoter of Rab20 are highlighted by a blue box.

***IKZF1* and RAB20 Expression are Correlated in Patients with B-ALL**
(Metanalysis of GEO data contributed by ML den Boer & JM Boer)

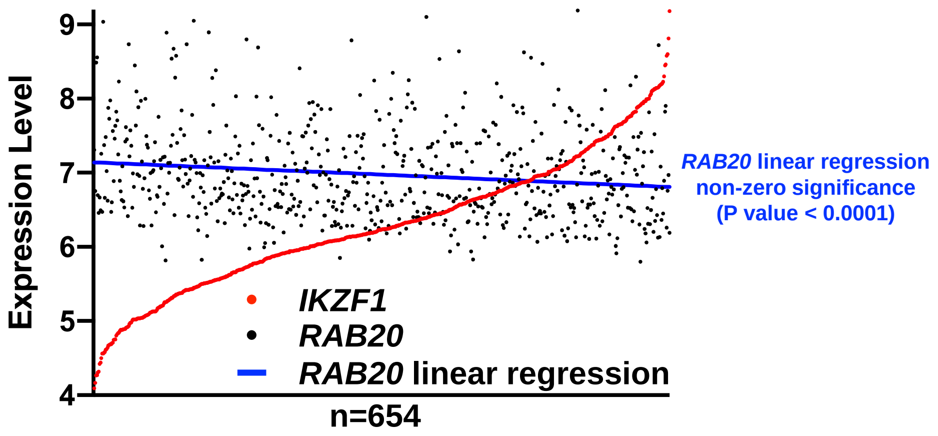

Supplemental Figure 2. *RAB20* expression negatively correlates with Ikaros expression in B-ALL. In a metanalysis of B-ALL patients (n=654), patients were ranked by increasing *IKZF1* expression level. *RAB20* expression for these patients was also plotted. Linear regression of *RAB20* expression revealed an inverse correlation between *RAB20* expression and *IKZF1* expression (slope = -0.0005098) that was significantly non-zero (P value < 0.0001).
